# Methodological considerations in using patient reported measures in dialysis clinics

**DOI:** 10.1186/s41687-017-0010-9

**Published:** 2017-11-05

**Authors:** John D. Peipert, Ron D. Hays

**Affiliations:** 1 Division of Nephrology, David Geffen School of Medicine, University of California, Los Angeles, 1018 Westwood Blvd, Suite 1223, Los Angeles, CA 90024 USA; 2 Terasaki Research Institute, Los Angeles, CA USA; 3 Division of General Internal Medicine and Health Services Research, David Geffen School of Medicine, University of California, Los Angeles, California, Los Angeles USA

**Keywords:** Dialysis, Outcomes, Patient reported measures

## Abstract

Patient reported measures (PRMs), including patient-reported outcomes, play a critical role in dialysis care. The usage of PRMs is extensive in dialysis clinics. While there are excellent PRMs to choose from, and their implementation as part of quality improvement and performance monitoring is extensive, there are still methodological challenges to be addressed. In this paper, we identify key methodological concerns around use of PRMs in dialysis centers in the United States and make recommendations for improving the use of PRMs in dialysis related to Selection of PRMs, Mode of Administration, and Support for PRM Use. These recommendations include: (1) Continue the use of Kidney Disease Quality of Life 36-item survey (KDQOL™-36) for dialysis centers’ internal quality improvement activities and the In-Center Hemodialysis Consumer Assessment of Health Care Providers and Systems (ICH-CAHPS survey®) for public dialysis center performance monitoring, but promote efforts to modify these instruments by incorporating PROMIS general health items (KDQOL-36) and reducing the length of the ICH-CAHPS. (2) Adopt a PRM of whether dialysis patients have been informed about all dialysis and transplant options. (3) Evaluate equivalence between electronic and paper versions of PRMs prior to widespread use of electronic administration. (4) Explore reimbursement of costs of PRM administration by the Centers for Medicare and Medicaid Services and kidney organizations. (5) Continue development of provider trainings in PRM administration and interpretation. These recommendations will help dialysis care decision-makers, clinicians, and applied researchers take the next steps toward enhancing PRM use in dialysis.

## Background

When a patient’s kidneys fail, renal replacement therapy is required to prevent death. Of the 678,000 patients with kidney failure in the United States (U.S.), 70% of them are on dialysis [1]. Most use hemodialysis, where a machine is used to filter wastes from the patient’s blood. Otherwise, patients may use peritoneal dialysis, where a peritoneal cavity in the patient’s abdomen is used to store and filter wastes from the blood. Both forms of dialysis are burdensome to patients and must be taken several times per week. In particular, most patients receive dialysis in clinics, though a minority of patients are dialyzed at home.

Patient reported measures (PRMs), including patient reported outcomes (PROs), play a critical role in medical care as a source of information about clinical experience and outcomes of care for patients, and dialysis is no exception [2]. For this paper, we adopt the FDA definition of a PRO: “any report coming from patients about a health condition and its treatment, without interpretation of the patient’s response by a clinician or anyone else” (p. 2) [3]. Though this definition was intended to apply specifically to PROs, we find it appropriate for all PRMs, with PROs being a subset of PRMs. In the field of dialysis, PRMs are used as performance measures. Since the Centers for Medicare and Medicaid Services (CMS) covers the cost of patient care for most renal replacement therapy, an extensive effort has been made to track patient experiences with care and health-related quality of life (HRQOL), with large data collection projects funded by government agencies. If dialysis centers have not demonstrated that the mandated PRM assessment has occurred, their reimbursement from CMS is in jeopardy.

The need for PRM measurement with dialysis patients cannot be understated. First, HRQOL may be suboptimal for dialysis patients. Dialysis is associated with many side effects and impacts on physical health. For example, in a study comparing the HRQOL of many chronic and infectious conditions (AIDS, epilepsy, gastroesophageal reflux disease, prostate disease, depression, diabetes, end-stage renal disease, multiple sclerosis), patients with end stage renal disease on dialysis had among the lowest physical functioning scores on the SF-36; only patients with multiple sclerosis had worse physical functioning [4]. In addition, dialysis may have a significant, negative impact on mental health [5]. In addition to HRQOL, it is critical to assess patient experience with dialysis care. Since dialysis is required multiple times per week, patients spend several hours in clinics and with their providers. With this in mind, CMS has included experience with care as part of its algorithm for determining the quality of dialysis center performance.

Though there has been significant uptake of PRMs in dialysis, several methodological issues known to impact of the success of PRM administration should be addressed. Therefore, in this report, we comment on key methodological issues around PRM administration in dialysis centers in the United States. We focus on which measures are available for use with dialysis patients, how PRMs can be used in clinical dialysis settings, and provide recommendations for overcoming challenges in administering PRMs. Within this discussion, we also comment on how current PRMs may be used as performance measures. The anticipated audiences for this report are dialysis medical providers and applied researchers who seek to use PRMs with dialysis patients, as well as dialysis care regulators (e.g., CMS), payors (insurance companies), and policy makers.

## Identifying patient-reported measures for dialysis patients

**Fig. 1:**
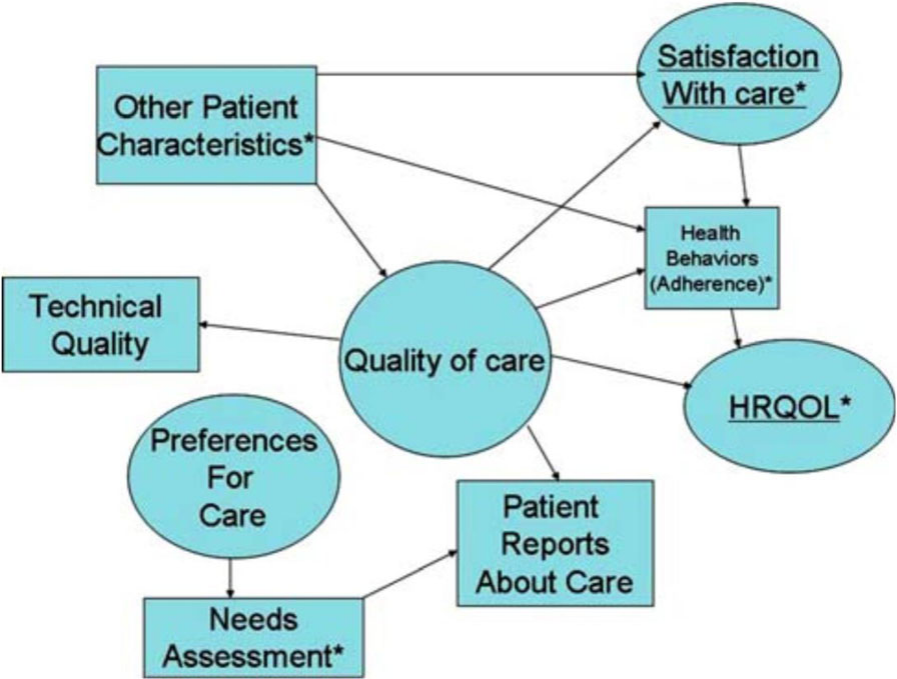
Fung and Hays Conceptual Model for Patient Reported Measures

It is critical to select PRMs that elicit information about dialysis patients’ use of and experience with treatments, as well as the outcomes of those treatments. Fung and Hays offered a framework that identifies PRMs appropriate for use across the trajectory of a treatment course and articulates the relationships between types of PRMs, and this framework is useful for identifying PRMs to use with dialysis patients [2]. (Fig. 1) Major PRMs in this framework include preferences for care, HRQOL, patient reports about their experiences with care, quality of care, and satisfaction with care. Experience with care refers to objective dimensions of the care patients receive and interactions with different elements of the health care system [6]. Satisfaction with care regards discrepancies between patients’ expectations for care and the care they actually receive [6]. Satisfaction with care and HRQOL are considered PROs, while other PRMs in the framework are not.

### Health related quality of life measures for dialysis patients

Recent reviews of PROs in kidney disease have obviated the need for a comprehensive review in this paper [7, 8]. Instead, we choose to focus on measures we recommend for use with dialysis patients based on whether patient input was used in creation of the measure, coverage of both universal and disease-targeted assessment, psychometric properties, and ability to compare to clinically relevant normative scores.

**Fig. 2:**
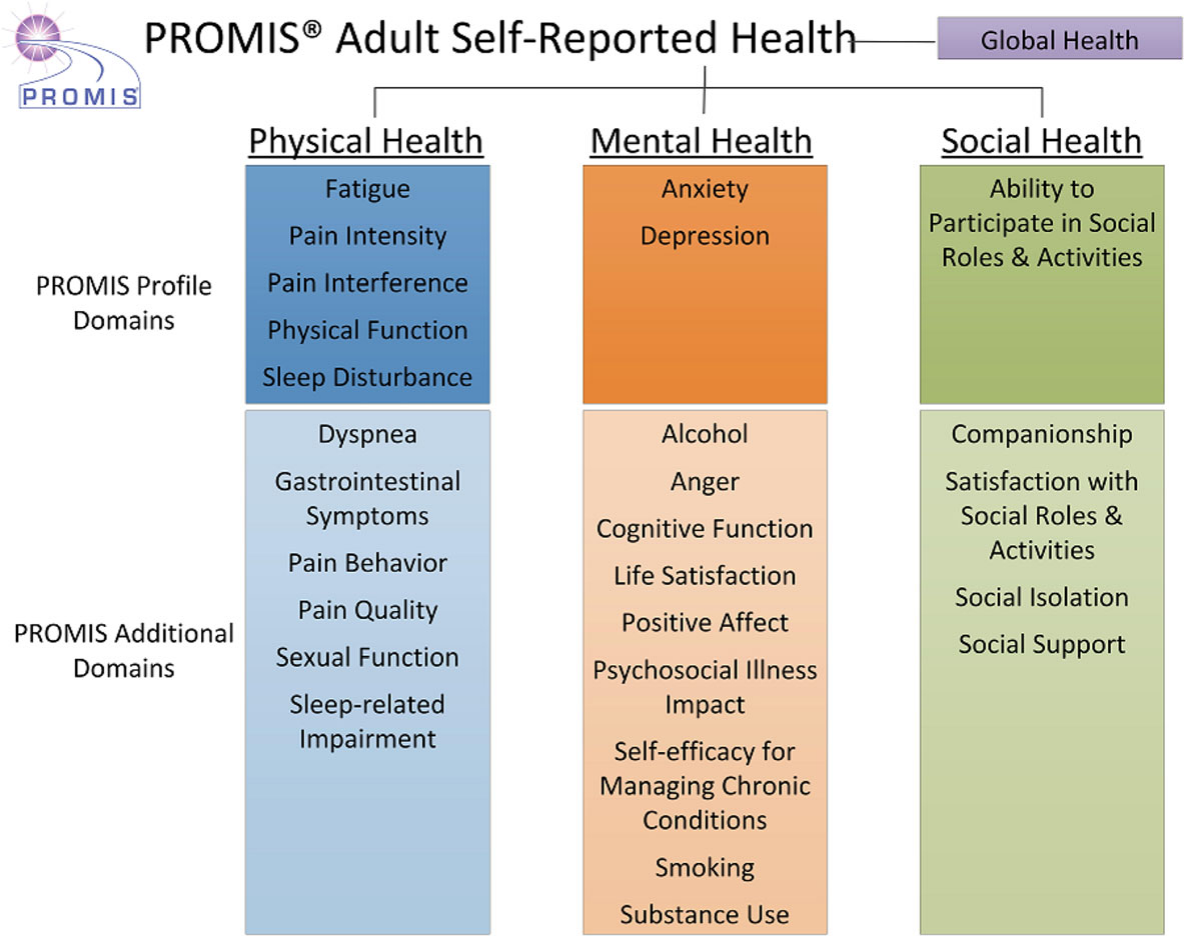
PROMIS Domain Framework for Health-Related Quality of Life

*PROMIS®.* The most frequently assessed PRO is HRQOL. The NIH PROMIS® project produced state-of-the-science HRQOL measures. PROMIS took an innovative approach to the development and evaluation of PROs by use of item response theory (IRT) and computer adaptive testing (CAT), drawing from large banks of items to generate efficient, reliable, and parsimonious individually-tailored measures of HRQOL [9]. Fig. 2 shows the PROMIS domain framework that features Global Health, Physical Health, Mental Health, Social Health, and several domains within these areas. PROMIS measures have demonstrated exceptional psychometric properties [9–11]. Of particular note, PROMIS measures have demonstrated superior reliability to legacy HRQOL measures across a range of patients’ underlying levels of HRQOL [12]. The PROMIS measures use a T-score metric, which has a mean of 50 and standard deviation of 10, referenced to the U.S. general population, which facilitates interpretation and comparison to nationally representative normative scores.

The PROMIS measures are universal. For this reason, PROMIS measures may be well-suited to capture the global aspects of HRQOL for dialysis, but may not fully reflect specific issues facing dialysis patients. Though there is increasing attention paid to the potential for PROMIS measures to be used in kidney disease [13, 14], these measures have not been systematically implemented with adult dialysis patients. It is worth noting that the PROMIS pediatric measures have begun to be implemented with pediatric ESRD patients [15, 16]. One limitation to use of the PROMIS measures includes their scoring referenced to the U.S., which may create barriers for applications outside the U.S.

#### Kidney disease quality of life (KDQOL) measures

When possible, it is recommended that a combination of universal and condition-targeted HRQOL measures be used, and there is evidence that kidney disease providers prefer assessments that include both types [8]. For example, the original KDQOL short-form (KDQOL-SF) includes the SF-36 as its generic core, supplemented by 11 kidney disease targeted domains (e.g., Symptoms/Problems of Kidney disease). The KDQOL-SF has demonstrated excellent reliability and validity, and is recommended for use with dialysis patients [8, 17]. A briefer version of the KDQOL-SF, the KDQOL-36 includes the SF-12 as the generic core and 24 additional items targeted at kidney disease (Symptoms/Problems, Effects of Kidney Disease, and Burden of Kidney Disease), and it too has demonstrated reliability and validity [18–21].

Considering the advantages and disadvantages of HRQOL measures used in dialysis, we recommend the continued use of the KDQOL-36 instrument with dialysis patients for the purposes of dialysis centers’ internal quality improvement. (Table 1) In addition to having attractive psychometric properties, the KDQOL-36 has been successfully applied to date with many thousands of dialysis patients, providing an unrivaled opportunity to compare individual patient scores to norms from the general dialysis population or from subgroups within this population. For these reasons, the KDQOL-36 was recently endorsed for inclusion in European renal registries in a consensus conference [8]. Despite these properties, we recommend, HRQOL by CMS for dialysis quality assessment, including KDQOL-36 scores. This issue has been debated in the literature [22, 23], but further research on the potential ramifications of using any HRQOL measure to rate dialysis center performance should be conducted before this strategy is pursued.

**Table 1:** Specific Recommendations for Continued Use of PRMs in Dialysis Centers

Category	Recommendations
Selection of PRMs	❖ Continue the use of Kidney Disease Quality of Life 36-item survey (KDQOL™-36) for dialysis centers’ internal quality improvement activities and the In-Center Hemodialysis Consumer Assessment of Health Care Providers and Systems (ICH-CAHPS survey®) for public dialysis center performance monitoring, but promote efforts to modify these instruments by incorporating PROMIS general health items (KDQOL-36) and reducing the length of the ICH-CAHPS. ❖ Adopt a PRM of whether dialysis patients have been informed about all dialysis and transplant options.
Mode of Administration	❖ Evaluate equivalence between electronic and paper versions of PRMs prior to widespread use of electronic administration.
Support for PRM Use	❖ Explore reimbursement of costs of PRM administration by the Centers for Medicare and Medicaid Services and kidney organizations. ❖ Continue development of provider trainings in PRM administration and interpretation.

*PRM* Patient reported measure

There are opportunities to improve the KDQOL-36. The incorporation of the SF-12 as the KDQOL-36’s universal HRQOL companion is no longer ideal. As described in the *PROMIS* subsection above, there have been major advancements in HRQOL measurement science, and the PROMIS measures now represent the state-of-the-science in universal HRQOL measures. Additionally, while the KDQOL-36 scales represent important dimensions of HRQOL for dialysis patients, they were developed over 20 years ago, and a changing dialysis population could signal the need for an update, or at least re-assessment, of kidney-targeted scales. Therefore, we recommend that a new version of the KDQOL-36 be developed with PROMIS measures as a generic core, and exploration of potential for fine-tuning among the kidney disease-targeted scales. An important caveat to this recommendation is that an approach should be taken such that new scores yielded from an updated version of the KDQOL-36 should be statistically linked to the original version. We do not recommend additional use of the PROMIS measures for mandated quality improvement or outcomes monitoring in dialysis centers. However, specific uses of any of the PROMIS measures, like research projects in which PROMIS-relevant domains are involved, are strongly recommended.

### Other types of patient reported measures for use with dialysis patients

In addition to the HRQOL measures described in the previous section, there are many other types of PRMs that play a critical role in understanding patients’ health and health care experiences in dialysis. Considering Fung and Hays’s framework (Fig. 1), other types of PRMs that need to be considered include patients’ health behaviors, preferences for care, patients’ experiences with care, and even patients’ decision-making characteristics about how they treat their kidney disease. There are multiple PRMs that fit these categories in use in research and in clinical practice, though there is significant opportunity to expand their use.

#### CAHPS in-center hemodialysis survey

The CAHPS In-Center Hemodialysis Survey was supported by the Agency for Health Research and Quality and CMS. CAHPS surveys are based on a definition of patient experience as “the range of interactions that patients have with the health care system, including their care from health plans, and from doctors, nurses, and staff in hospitals, physician practices, and other health care facilities” [24]. CMS has adopted several CAHPS measures for quality improvement in addition to ICH-CAHPS, including the CAHPS Hospital, Home and Community-Based Services, Hospice, Surgery, and Medicare ambulatory surveys. The ICH-CAHPS survey items are targeted at care provided to hemodialysis patients, and these items would not be appropriate for consumers of other types of health services. The ICH-CAHPS includes 3 composites: Nephrologists Communication and Caring, Providing Information to Patients, and Quality of Dialysis Center Care and Operations. Additionally, 3 other items provide global ratings of patients’ experience with their kidney doctors, dialysis center staff, and dialysis center. Support for the reliability and validity of these composites has been provided [25].

**Fig. 3:**
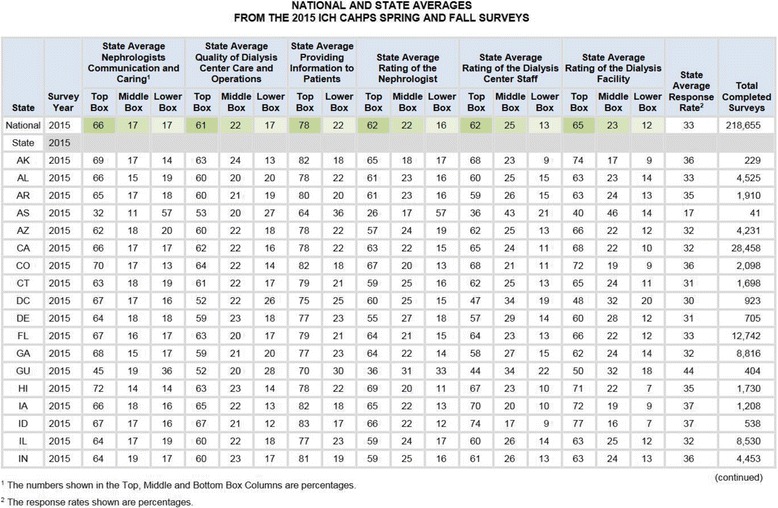
ICH-CAHPS 2015 National and State Average Scores

**Fig. 4:**
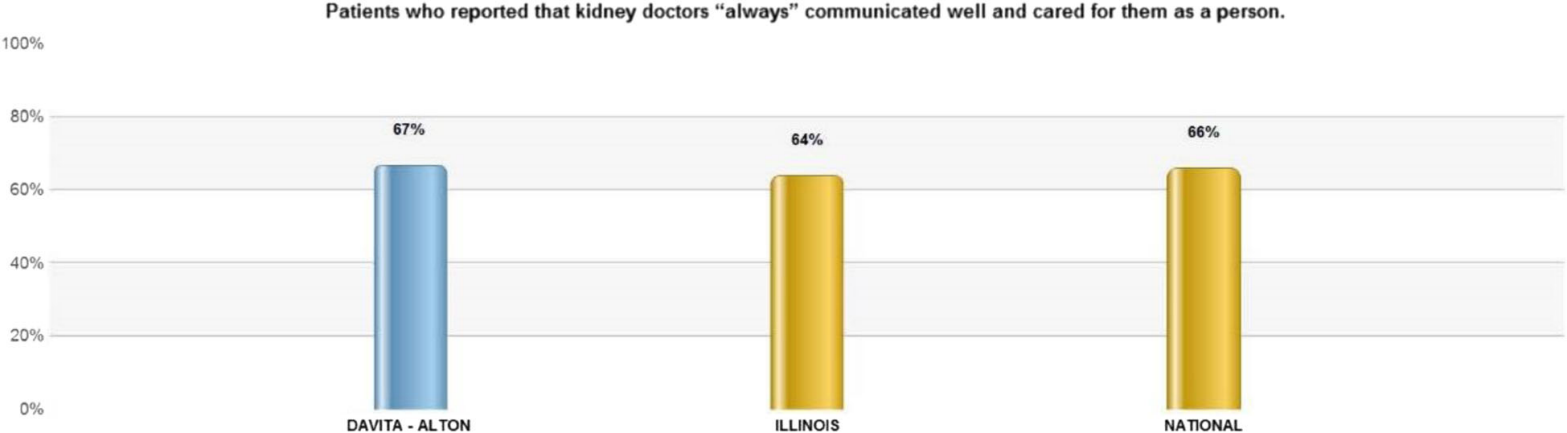
Example of ICH-CAHPS Items Comparisons from Dialysis Center Compare

Due to CMS’s use of ICH-CAHPS as a clinical measure in the payment year (PY) 2019 QIP, it is assessed twice yearly in all dialysis centers throughout the United States. Figure 3 shows examples of national and state averages of ICH-CAHPS surveys in 2015. The results of patients’ reports about different dialysis clinics with the ICH-CAHPS are available to view on CMS’s Dialysis Facility Compare website: https://www.medicare.gov/dialysisfacilitycompare/. These comparisons show differences between centers of interest, and to state and national averages. An example of comparison of a dialysis center to state and national norms is given in Fig. 4. Recent reports from CMS indicate that the ICH-CAHPS will continue to play a large role in dialysis service evaluation and figure into CMS’s ratings of dialysis center performance.

Given its attractive measurement properties and its ability to be used for comparisons among clinics and to state and national norms, we recommend the continued use of the ICH-CAHPS for CMS’s dialysis center performance monitoring. However, there are also opportunities to optimize this measure, especially to make it more parsimonious, reducing burden among patients and providers. A recent report detailed efforts to shorten the CAHPS Clinician and Group adult survey without significant reduction in reliability or clinically-important content [26]. These analyses showed that the Provider Communication and the Access scales could be reduced from 6 and 5 to 2 items, respectively. Noting the length of the ICH-CAHPS composites at 6 items (Nephrologists Communication and Caring), 9 items (Providing Information to Patients), and 17 items (Quality of Dialysis Center Care and Operations), the ICH-CAHPS is ripe for similar analyses.

#### Additional patient reported measures

There are several other PRMs relevant to dialysis patients. Commonly assessed health behaviors include diet and exercise regimen [27]; use of alcohol, tobacco, or other substances [27]; and adherence to medications [28], dialysis appointments [29] or other prescribed treatments. Similarly, preferences for care encompasses many patient preferences about use of health care like the degree of agency in their relationship and communication with providers [30] to preferences for end-of-life care [31]. Finally, and somewhat related to preferences for care, patient characteristics that influence patients’ decision-making about their care represent important PRMs and may include readiness for particular types of treatment (e.g, peritoneal dialysis), perceived benefits and costs of different treatments, self-efficacy to pursue different treatment options, and knowledge of treatment options [32].

Patients’ decision-making about their treatment is a particularly important domain where the use of PRMs should be expanded in clinical dialysis care. There are several types of dialysis and options for how the treatment is taken. Hemodialysis and peritoneal dialysis, and whether patients take dialysis in a center or at home, entail drastically different experiences for patients, and often have different outcomes [1, 33–35]. Additionally, dialysis is not the only treatment open to kidney patients, and CMS has required that patients have the opportunity to learn about kidney transplant. According to CMS’s 2008 Conditions for Coverage, dialysis centers must provide information about the option for kidney transplant to each dialysis patient, and indicate that they have done so on CMS Form-2728 at the time of initiation of chronic dialysis. In part, this requirement reflects the need to ensure that patients are able to make an informed decision, and therefore give informed consent, to their dialysis treatment. To date, this report on Form-2728 is made by the dialysis provider, but there is evidence that patients actually report being educated about transplant less-often than providers report educating them when data from Form-2728 are compared to those from patient surveys [36]. Studies like this indicate that incorporation of patient reports about whether they have received adequate education for their treatment options, along with reports about their preferences, may be better indicators of whether informed decision-making and consent around treatment choices actually occur among dialysis patients. Therefore, we recommend the adoptions of a PRM of whether patients have been informed about all their dialysis and transplant options.

### Approaches to administering patient reported measures in dialysis centers

Given the importance of PRMs to understanding patients’ health and experiences with health care, it is often advantageous to include them in interventions in clinical settings such as dialysis centers. The International Society for Quality of Life Research (ISOQOL) put forth guidance for incorporation of PRMs in clinical care, which has extensive relevance for administering PRMs to dialysis patients [37, 38]. In doing so, they use Greenhalgh and colleagues’ taxonomy, which makes the following recommendations for implementing PRMs in clinic [39]. First, PRMs are used to screen for health problems. Once health problems are identified, PRMs are used to monitor those problems over time. Finally, clinicians need PRM information to facilitate shared decision-making about treatment.

Additionally, Santana and Feeny [40] created a framework to guide the application of PRMs in routine clinical care, which includes the following components: 1) communication between patients, their support networks, and providers; 2) patient engagement in the care process; 3) shared decision-making; 4) patient management (self-management and provider management); 5) patient satisfaction; 6) provider satisfaction; 7) patient adherence to treatments; and 8) patient outcomes (e.g., HRQOL). Each of these components represents a dimension of clinical care that will be improved by the application of PRMs.

Major aspects of the ISOQOL and Santana and Feeny frameworks have been implemented for routine PRM assessment in dialysis centers. As noted above in the Background section, in their Conditions for Coverage (42 CFR §494.90), CMS mandated that each dialysis patient’s physical and mental health must be monitored, and this often occurs with the use of a standardized HRQOL measure [41]. The patient reports of HRQOL are then used to create individually-tailored interventions that focus on the areas where the patient’s HRQOL needs most improvement. This approach essentially employs the ISOQOL recommendations. To date, little is known about the impacts of this process on improved outcomes for patients with kidney disease and communication between patients and providers. Interventional studies should examine this impact. Another way to improve PRM administration and further align with the ISOQOL framework involves our recommendation (see below in the *Timing of Patient Reported Measures in Dialysis* subsection of this paper) that HRQOL assessment be increased to a bi-annual basis to generate more consistent monitoring of improvements in health, or lack thereof, resulting from the tailored care plans.

In addition, as we noted in the Background section, reports of patient experience with care using the CAHPS In-Center Hemodialysis survey are included in the Quality Incentive Program (QIP) evaluation metrics for dialysis centers [42]. This requirement incorporates important aspects of the Santana and Feeny framework. Most notably, ICH-CAHPS assessments increase patient engagement in the care process by providing feedback to providers on how their care is going. Providers, dialysis administrators, and other patients can use this information to improve the quality of care, as well as to help patients make decisions about which clinic to receive their dialysis care. Use of ICH-CAHPS may also improve communication by creating an opportunity for patients to report on negative aspects of their care they may not feel comfortable discussing in face-to-face meetings with providers [43]. One element of the Santana and Feeny framework currently not addressed by PRM mandates in dialysis regards provider satisfaction. Dialysis providers, as well as staff members (e.g., nurses, social workers, technicians) have an important perspective to share about how to improve dialysis care, and a more formal mechanism for eliciting those perspectives would be beneficial. Previous measurement systems for complementary assessments of patient and provider satisfaction with care could serve as the basis for this effort [44].

The remainder of this section of the paper will be dedicated to more practical aspects of PRM administration in dialysis centers, including mode of administration and timing of PRMs. These issues have received insufficient attention in the dialysis literature and require future examination.

#### Mode of patient reported measure administration

There are several ways to administer PRMs with dialysis patients. As the use of PRMs in dialysis clinics has expanded, clinicians and researchers have attempted to identify the best ways to administer these measures. In 2015, ISOQOL conducted a comprehensive assessment of the resources needed and tradeoffs associated with different modes of administration of PRMs [38]. (Table 2) Within the clinic, surveys can be self-administered, interview-administered, or computer administered. On the phone, the concentration is on interview administration or administration through an automated, voice-activation survey system. By mail, surveys are strictly self-administered by the patient and returned by mail. Similarly, on the web, surveys are self-administered by patients. Each of these modes requires specific resources that have implications for their feasibility. Successfully conducting surveys with any of these methods requires consideration of needs for staff, technology, and potentially informatics infrastructure, and this can be expensive.

**Table 2:** International Society for Quality of Life Research Summary of Mode of Administration for PRMs

	Resources Needed	Advantages	Disadvantages
In-Clinic			
Self Admin.	• Personnel to supervise and assist • Space • Personnel for data entry	• Low-technology requirements • Implemented in any clinical setting • Relatively low cost	• Problem with low literacy patients & visual handicap • Difficult with other special populations (e.g., very young, very old) • Higher rate of missing data
Interview Admin.	• Skilled interviewer • Space • Personnel for data entry	• More personal • In-depth questioning • No issues with literacy and/or visual handicap	• Relatively expensive • Social desirability bias • Staff time
Computer Admin.	• Personnel to supervise and assist • Software to collect & report data	• Efficient data capture and entry	• Problems finding space/providing privacy • Costs to obtain & maintain PRM system • Potential software problems
Mail			
Self Admin.	• Personnel to manage mailing • Personnel for data entry	• Low-technology requirements • Potentially simpler logistics than in-clinic administration • Relatively low cost	• High non-response rate • Cannot ensure patient completes questionnaire alone • Hard to respond immediately to patient needs • Challenges scheduling assessment near clinical visit • Other limitations similar to Self-Administered In-Clinic
Telephone			
Interview Admin.	• Skilled interviewer • Personnel for data entry	• More personal • More convenient for patient • Largely circumvents literacy problem and/or visual handicap	• Lack of visual cues as compared to face-to-face • Relatively expensive • Potential problem with social desirability • Some topics may be more difficult to address
Voice Activated	• Personnel to oversee data collection • Validated interactive voice response (IVR) system	• Low cost due to automation	• May not be accepted by patients • Costs to obtain & maintain IVR system • Requires process to track and respond to any urgent problem reported by patients • Other disadvantages similar to Live Telephone Interview, plus impersonal nature
Web-Based	• Systems management personnel • Software to collect and report the PRO data • Training patients	• Efficient data capture with simultaneous data entry • Convenient for patient • Flexible timing for data collection	• Difficult to ensure privacy • Upfront costs for the PRO system and maintenance • Potential software problems

Reprinted from Aaronson N, Choucair A, Elliott T, et al. User’s Guide to Implementing Patient-Reported Outcomes Assessment in Clinical Practice. International Society for Quality of Life Research; 2015

The potential efficiencies of electronic survey administration methods have seen this approach grow in recent years. One particular benefit accrued in web-based surveys is the direct input of data into a database that can be immediately sourced for analysis. Through this approach, the need for data entry into a database is eliminated, which is attractive not only because it reduces the amount of personnel and time dedicated to administering PRMs, but also because it may reduce data entry error. Once data is entered through a web interface, the ease of integrating these data within the electronic medical record is increased as well. Given that often the ultimate objective of collecting PRMs is to combine them with other important clinical data, the ability to do so with ease and in real time is a considerable benefit.

One example of electronic data entry platforms for PRMs in dialysis clinics is found in the Medical Education Institute’s (MEIs) administration of the KDQOL-36. The MEI uses the KDQOL-36 in dialysis centers throughout the United States as part of their KDQOL-Complete program. The KDQOL-Complete program helps meet the CMS requirement to create individualized care plans for each dialysis patient. The KDQOL-Complete program assesses dialysis patients using the KDQOL-36 and tailors care plans for each patient in order to improve aspects of HRQOL that are below expectations. The ability to enter data electronically into the KDQOL-Complete computerized platform allows for automated scoring of the KDQOL-36 so that individual patients’ scores can be viewed immediately. Additionally, this computerized system can compare an individual patient’s scores to national norms and generate illustrative graphics to help the dialysis provider and patient understand the scores.

Despite these benefits, some considerations for the integrity of electronic PRM administration should be made before pursuing this strategy. Many instruments were designed for paper/pencil [45]. PRMs often do not need to be completely redeveloped for electronic administration, but additional testing for equivalence should be conducted, leading to some instrument modification [45]. The types of changes needed range from relatively minor to extensive. Examples of small changes include things like updates to instructions and formatting. Examples of moderate changes include things like updates to item wording. Examples of significant changes include substantial changes to item wording or response options. Empirical studies suggest little difference between electronic and paper and pencil versions of instruments [46, 47]. Currently, there is a need for studies on key PRMs used in dialysis to determine if any modifications are needed before electronic administration is advised. Therefore, we recommend that studies be conducted to evaluate equivalence between electronic and paper versions of PRMs before widespread use of electronically administered PRMs in dialysis.

#### Timing of patient reported measures in dialysis

The appropriate timing of PRM administration in clinic depends on the purpose of the assessments. The FDA’s Center for Drug Evaluation and Research identified the following possible assessment approaches: at the beginning and end of a particular treatment, at or after important medical events occur (e.g., starting a new type of dialysis, development of a clinically significant comorbidity), or at regular, repeated intervals to examine progress of a chronic condition [48, 49]. Though all of these approaches may be relevant to PRM assessment as part of standard clinical care in dialysis, long term monitoring over repeated intervals may be most relevant. For instance, a HRQOL measure may be assessed when a patient begins dialysis then could be administered at regular intervals thereafter to track potential improving or worsening health.

Indeed, the most benefit may be realized from administering PRMs at multiple occasions with patients. Multiple administrations allow clinicians to track changes over time to monitor disease progression or to examine responses to changes in treatments. Despite this benefit, most often, PRMs are infrequently administered to patients. This is also the case within the field of dialysis as well. Although CMS has mandated the assessment of HRQOL and patient experience, these PRMs are most often assessed only once or twice for each patient. While single timepoint assessments are certainly superior to no PRM assessment at all, repeated assessments drastically increase the capacity to understand patients’ health and experiences with dialysis so that adjustments in care can be made. The value of longitudinal assessment of PRMs has been raised specifically in regards to the CAHPS measures. CAHPS items ask about experiences with care over the previous 6 months; if repeated every 6 months, experiences with care occurring over specific durations of time can be isolated, and the reports generated can be easily used for quality improvement if necessary. Other approaches, wherein all care received in the past is asked about, do not offer the opportunity for longitudinal assessment, since repeated assessments of such measures would yield uninterpretable information due to the inability to pinpoint which specific durations of care were being described by the reports.

There are a few challenges to longitudinal assessment of PRMs. First, repeated measures on the same patient over time may entail a lack of timely reports of results to providers. Second, due to frequent patient turnover in some clinics, longitudinal measurement may not be possible for all patients, creating a potential challenge to standardized longitudinal measurement for all patients. Finally, many of the barriers to cross-sectional PRM measurement may be compounded with repeated measurement, including increased costs, provider burden, and patient burden. Considering both the benefits and burdens of repeated PRM measurement, we recommend that key PRMs be assessed twice annually for each patient so that some change can be observed in response to changes in treatment plans. The CMS requirements for the ICH-CAHPS already adhere to this recommendation, though we suggest that the KDQOL-36 assessment recommendations be altered so that it is also assessed twice annually.

#### Support for use of patient reported measures in dialysis

Though there are many clear benefits to administering PRMs in dialysis centers, there are also challenges. There is evidence that dialysis providers and staff often have an extensive workload, and adding of PRMs adds to this workload. A related practical challenge regards the additional cost associated with administering PRMs in the dialysis clinic. The staff time and resources, as well as material costs, required to administer a PRM, along with entering the data, then interpreting the results and incorporating the learning into clinical practice is not free, and may be difficult to justify in clinics without significant discretionary spending [2]. These challenges may be especially severe in centers with low staff-to-patient ratios. We recommend that new explorations be launched to identify mechanisms for ESRD Networks, CMS, and other kidney-focused organizations to reimburse these costs.

Another important barrier regards staff training to administer key PRMs [2]. When PRMs are administered in an interview style from the dialysis staff, they require understanding of standardized survey administration techniques, including ways to elicit unbiased, accurate responses and trouble shoot when patients have questions, understand potentially complex skip-patterns, and screen for patient responses that may be untruthful or not genuine (e.g., a patient gives several of the same responses consecutively quickly in order to complete the assessment). Even when PRMs are administered through self-administered surveys (e.g., mailed to the patients), data entry protocols to reduce error are recommended, and these require training. Therefore, we recommend the continued development of effective, low-cost training programs to help providers administer PRMs, including e-learning programs.

## Conclusions

In conclusion, there is a lot to celebrate in the field of PRMs in dialysis. Many strong measures have been developed and evaluated, and their use in dialysis centers is extensive. Despite these successes, there is significant room for improvement. We have identified several pointed recommendations for improving the use of PRMs in dialysis. There are a few limitations to consider regarding these recommendations. Primarily, this paper does not reflect a systematic, structured review of the literature. Secondarily, we have focused only on the topics we judged to be most germane to overcoming methodological issues in PRM administration in clinical settings, and does not consider their use in research efforts. These limitations withstanding, we have confidence that these recommendations will help dialysis care decision-makers and clinicians continue to improve the excellent track record of PRM use in dialysis.
